# Diet in subjects with irritable bowel syndrome: A cross-sectional study in the general population

**DOI:** 10.1186/1471-230X-12-61

**Published:** 2012-06-07

**Authors:** Solveig C Ligaarden, Stian Lydersen, Per G Farup

**Affiliations:** 1 Department of Medicine, Innlandet Hospital Trust, Kyrre Grepps gt 19, 2819, Gjøvik, Norway; 2 Unit for Applied Clinical Research, Department of Cancer Research and Molecular Medicine, Norwegian University of Science and Technology, Trondheim, Norway; 3 Regional Centre for Child and Adolescent Mental Health (RBUP), Department of Neuroscience, Norwegian University of Science and Technology, Trondheim, Norway; 4 Department of Research, Innlandet Hospital Trust, Gjøvik, Norway

**Keywords:** Human, Adult, Irritable bowel syndrome, Cross-sectional studies, Diet, Gastrointestinal tract

## Abstract

**Background:**

Patients with irritable bowel syndrome (IBS) often relate symptoms to the intake of certain foods. This study assesses differences in diet in subjects with and without IBS.

**Methods:**

The cross-sectional, population-based study was conducted in Norway in 2001. Out of 11078 invited subjects, 4621 completed a survey about abdominal complaints and intake of common food items. IBS and IBS subgroups were classified according to Rome II criteria.

**Results:**

IBS was diagnosed in 388 subjects (8.4%) and, of these, 26.5% had constipation-predominant IBS (C-IBS), 44.8% alternating IBS (A-IBS), and 28.6% diarrhoea-predominant IBS (D-IBS). Low intake of dairy products (portions/day) (Odds Ratio 0.85 [CI 0.78 to 0.93], p *=* 0.001) and high intake of water (100 ml/day) (1.08 [1.02 to 1.15], p *=* 0.002), tea (1.05 [1.01 to 1.10], p *=* 0.019) and carbonated beverages (1.07 [1.01 to 1.14], p *=* 0.023) were associated with IBS. A lower intake of dairy products and a higher intake of alcohol and carbonated beverages were associated with D-IBS and a higher intake of water and tea was associated with A-IBS. In subjects with IBS the severity of symptoms was associated with a higher intake of vegetables and potatoes in subjects with C-IBS, with a higher intake of vegetables in subjects with A-IBS, and with a higher intake of fruits and berries, carbonated beverages and alcohol in subjects with D-IBS.

**Conclusions:**

In this study, the diet differed in subjects with and without IBS and between IBS subgroups and was associated with the severity of symptoms.

## Background

Functional gastrointestinal disorders are common in developed countries, and irritable bowel syndrome (IBS) is the most frequent disorder with a prevalence of 5–10% [1]. IBS generates a considerable workload and constitutes 36% of all visits to gastroenterologists [2]. The treatment is not adequate.

Approximately two-thirds of subjects with IBS relate their symptoms to their intake of food [3,4]. Most of these subjects modify their diet, and these modifications sometimes result in an inadequate diet [3]. On the other hand, some subjects with IBS may not be aware of all the offending items. Despite reports of symptoms related to food intake, investigations of food intake in IBS have been sparse. One study conducted in the general population reported no differences in the consumption of specific food items between subjects with functional gastrointestinal disorders and controls [5], while another study, conducted in secondary care, reported the consumption of a poorer quality diet by subjects with IBS [6]. Except for the previously mentioned population-based study, the diet of subjects with IBS in the general population has to our knowledge not been investigated. We hypothesised that diet was associated with IBS, IBS subgroups and severity of symptoms in a general population-based sample. Therefore, the primary aim of the present study was to assess differences in the diets of subjects with and without IBS. The secondary aims were to assess differences between IBS subgroups and between IBS subgroups and the population without IBS, and to assess associations between the severity of symptoms and diet within the IBS-population.

## Methods

The OPPHED (Oppland and Hedmark counties) Health Study was conducted in 2000 - 2001 as a cross-sectional study by the National Health Screening Service (now the Norwegian Institute of Public Health). In this part of the study, all men and women living in Oppland County who were born in 1925, 1940, 1955, 1960 and 1970 were invited to participate.

### Assessments

Subjects were asked to complete questionnaires in paper form on their own. The food and drink questions in our study were equal to the food and drink questions in The Oslo Health Study (an English translation is available [7]). The following information was derived from the answers to the questions: demographics, common diseases (asthma, bronchitis, diabetes, osteoporosis, fibromyalgia, mood disorders, heart attack, angina, cerebral stroke) (number of diseases, score 0-9), mood disorders measured by Hopkins Symptom Checklist (HSCL10) (score 1.0-4.0, mental distress ≥ 1.85), musculoskeletal complaints (score 0-12), smoking habits, activity habits, diet, and gastrointestinal symptoms. IBS was defined according to the Rome II criteria [8] and IBS subgroups were classified as constipation-predominant IBS (C-IBS), alternating IBS (A-IBS), or diarrhoea-predominant IBS (D-IBS). The severity of symptoms (score 1-12) was calculated as the product of severity (mild, moderate, severe (score 1-3)) and frequency (one day or less per week, two to three days per week, four to five days per week, more than five days per week (score 1-4)) The diet was assessed using a limited food frequency questionnaire (FFQ), and questions included the frequency and quantity of the intake of beverages (milk, water, carbonated beverages, and alcoholic beverages), fruits, vegetables, fatty fish, cheese, and omega-3 fatty acid supplements, but did not include the food groups cereals and meat. For the analyses, dairy product portions were classified as either 150 ml milk/yoghurt or 20 g (one slice) of cheese. Body mass index (BMI) was calculated by means of measured weight and height. Diastolic and systolic blood pressure and pulse were measured. Blood samples were taken and standard blood tests were performed (cholesterol, high-density lipoprotein (HDL), triglycerides, glucose, creatinine clearance). Blood levels of omega 3 fatty acids (22:6(n-3) + 22:5(n-3) + 20:5(n-3)) from fatty fish and fish oil supplements were measured in 60 subjects with IBS and in 60 controls. The method used was described for the first time by Bonaa et al. [9].

### Statistical methods

Data were analysed with PASW Statistics 18.0 (SPSS, Chicago, Illinois, USA).

Differences between subjects with and without IBS were assessed with Mann-Whitney *U* test, Chi-square test, and logistic regression analyses. Differences between the three subgroups of IBS were assessed with the Kruskal-Wallis Test and Chi-square test, followed by pairwise comparisons when the overall test was significant. Logistic regression analyses were used to assess differences between each of the three IBS subgroups (C-IBS, A-IBS, and D-IBS) versus subjects without IBS. Associations between the severity of symptoms and IBS subgroups were assessed with ordinal logistic regression analyses. In situations with fewer than 10 observations per covariate, we used backward stepwise elimination terminating in models that included age, gender, HSCL10, and musculoskeletal complaints, and as many other covariates as possible with at least 10 cases per covariate.

For the regression analyses, missing values were handled by multiple imputations. All variables to be included in the regression analyses were included in the imputation model. Right-skewed variables were log-transformed before being used in the imputation model and 20 datasets were created.

Two-sided p < 0.05 was considered statistically significant, and 95% confidence intervals (CI) are reported where relevant. All results are given as the mean with the SD in parentheses unless otherwise indicated.

### Ethics

All participants gave written informed consent to participation before enrolment in the study. The project was approved by the Regional Committees for Medical Research Ethics, and the Data Inspectorate, Oslo, Norway.

## Results

**Figure 1:**
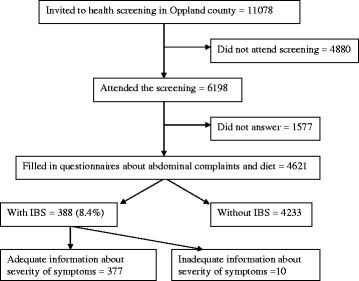
Flow chart.

Questionnaires about abdominal complaints and diet were completed by 4621 subjects (Figure 1). Of the eligible subjects, 388 (8.4%) men and women were diagnosed with IBS, and of these, 26.5% had C-IBS, 44.8% A-IBS, and 28.6% D-IBS. The mean (SD) severity of their symptoms was 3.2 (2.4).

Subjects with IBS had significantly lower intake of dairy products and potatoes and significantly higher intake of water, tea and carbonated beverages than subjects without IBS (Table 1). The population with IBS had a significant higher score for number of diseases, mood disorders and musculoskeletal complaints compared with controls.

**Table 1:** Subject characteristics and dietary intake in subjects with and without IBS

**Subject characteristics and food groups (% missing)**	**IBS n = 388**	**Non IBS n = 4233**	**p**
Female, % (0.0)	67.5	55.3	< 0.001
Age, years (0.0)	46.9 (13.4)	49.1 (13.9)	0.002
BMI, kg/m^2^ (0.2)	26.9 (4.5)	26.9 (4.2)	0.58
Education, years (1.0)	12.0 (3.5)	12.2 (3.5)	0.67
Number of diseases (0-9) (5.7)	1.0 (1.3)	0.5 (0.9)	< 0.001
HSCL10^1^ (1-4) (6.4)	1.6 (0.6)	1.3 (0.4)	< 0.001
Musculoskeletal complaints (0-12) (28.9)	3.6 (2.9)	1.9 (2.1)	< 0.001
Physical activity, hours/day (9.3)	2.8 (1.7)	3.0 (1.7)	0.020
Smoking, % (0.6)	32.7	27.9	0.045
Cholesterol, mmol/L (0.1)	5.6 (1.1)	5.7 (1.1)	0.036
HDL, mmol/L (0.1)	1.3 (0.4)	1.4 (0.4)	0.061
Triglycerides, mmol/L (0.2)	1.6 (1.0)	1.7 (1.1)	0.12
Glucose, mmol/L (0.5)	5.3 (1.3)	5.4 (1.4)	0.39
Creatinine clearance (0.2)	88.1 (25.8)	87.7 (25.5)	0.80
Systolic blood pressure, mm Hg (0.0)	127.1 (20.7)	130.3 (20.9)	0.002
Diastolic blood pressure, mm Hg (0.0)	73.2 (10.2)	74.4 (10.2)	0.028
Pulse, beats/minute (0.1)	72.5 (11.2)	71.9 (12.4)	0.23
Fruits and berries, g/day (1.3)	86.0 (74.8)	92.9 (76.0)	0.035
Vegetables, g/day (1.6)	150.5 (116.0)	147.4 (108.9)	0.87
Potatoes, g/day (0.7)	123.5 (83.4)	136.3 (88.0)	0.005
Dairy products, portions^2^/day (13.8)	2.2 (1.5)	2.6 (1.5)	< 0.001
Water, 100 ml/day (1.1)	3.9 (1.9)	3.5 (1.9)	0.001
Carbonated beverages, 100 ml/day (0.7)	1.8 (2.1)	1.4 (1.6)	<0.001
Coffee, 100 ml/day (0.6)	4.2 (3.8)	4.4 (3.3)	0.16
Tea, 100 ml/day (0.4)	1.7 (2.5)	1.3 (2.3)	0.003
Juice, 100 ml/day (5.2)	0.9 (1.2)	0.8 (1.0)	0.37
Alcohol, units^3^/day (4.1)	0.22 (0.50)	0.19 (0.29)	0.12
Omega 3 fatty acids^4^, g/day (4.3)	0.7 (0.7)	0.8 (0.7)	0.012

^1^ Hopkins Symptom Checklist 10 for mood disorders measurement.

^2^ Dairy product portions are defined as either 150 ml milk/yoghurt or 20 g (one slice) of cheese.

^3^ Units of alcohol are defined as 12.8 g (15 ml) of pure alcohol.

^4^ Omega 3 fatty acids are calculated from fatty fish and fish oil supplements.

From original data with Mann-Whitney *U* test/Chi-square test. Values are mean (SD) unless otherwise stated.

A comparison of IBS subgroups showed a significant higher intake of alcohol in subjects with D-IBS and A-IBS compared to subjects with C-IBS (Table 2). There were significantly more men with D-IBS. Moreover, the severity of symptoms was significantly higher among those with D-IBS compared to the other subgroups.

A comparison of IBS subgroups showed a significant higher intake of alcohol in subjects with D-IBS and A-IBS compared to subjects with C-IBS (Table 2). There were significantly more men with d-IBS. Moreover, the severity of symptoms was significantly higher among those with D-IBS compared to the other subgroups.

**Table 2:** Subject characteristics and dietary intake: comparison between subgroups of IBS

**Subject characteristics and food groups**	**C-IBS n =103**	**A-IBS n =174**	**D-IBS n =111**	**p**
Age, years	48.3 (14.2)	46.0 (13.2)	47.0 (12.7)	0.51
Female, n, %	86 (83%)	123 (71%)	53 (48%)	<0.001^A^
Severity of symptoms (1-12)	2.7 (2.3)	3.0 (2.3)	3.8 (2.5)	<0.001^B^
Fruits and berries, 100 g/day	1.0 (0.7)	0.8 (0.7)	0.8 (0.8)	0.068
Vegetables, 100 g/day	1.7 (2.2)	1.5 (1.1)	1.4 (1.2)	0.10
Potatoes, 100 g/day	0.4 (0.9)	1.2 (0.8)	1.2 (0.8)	0.13
Dairy products, portions^1^/day	2.2 (1.5)	2.2 (1.6)	2.0 (1.4)	0.71
Omega 3 fatty acids^2^, g/day	0.7 (0.6)	0.7 (0.7)	0.7 (0.7)	0.87
Water, 100 ml/day	3.9 (1.9)	3.9 (2.0)	3.7 (1.9)	0.65
Juice, 100 ml/day	0.9 (1.2)	0.9 (1.1)	1.0 (1.2)	0.90
Carbonated beverages, 100 ml/day	1.4 (1.6)	1.9 (2.4)	2.0 (2.1)	0.13
Tea,100 ml/day	1.7 (2.2)	1.9 (2.6)	1.4 (2.6)	0.094
Coffee,100 ml/day	4.3 (3.5)	4.0 (4.2)	4.4 (3.3)	0.39
Alcohol, units^3^/day	0.1 (0.2)	0.2 (0.6)	0.3 (0.6)	0.006^C^

^1^ Dairy product portions are defined as either 150 ml milk/yoghurt or 20 g (one slice) of cheese.

^2^ Omega 3 fatty acids are calculated from fatty fish and fish oil supplements.

^3^ Units of alcohol are defined as 12.8 g (15 ml) of pure alcohol.

^A^ A-IBS vs. D-IBS: p < 0.001; C-IBS vs. D-IBS: p < 0.001; C-IBS vs. A-IBS: p *=* 0.020 ^B^ A-IBS vs. D-IBS: p *=* 0.001; C-IBS vs. D-IBS: p < 0.001.

^C^ C-IBS vs. A-IBS: p *=* 0.008; C-IBS vs. D-IBS: p *=* 0.003.

From original data with Kruskal Wallis Test/Mann-Whitney *U* test for continuous and Fisher exact test for dichotomous variables. Only age, gender, severity of symptoms and food groups are shown. Values are mean (SD) unless otherwise stated.

Multivariable analysis showed that a lower intake of dairy products and a higher intake of water, tea and carbonated beverages were associated with IBS (Table 3). Subjects with D-IBS had significantly lower intake of dairy products and significantly higher intakes of alcohol and carbonated beverages than subjects without IBS. Furthermore, there was a significantly higher intake of tea and water in subjects with A-IBS compared with subjects without IBS. There were no significant differences in diet between subjects with C-IBS compared to those without IBS.

**Table 3:** Subjects with IBS, subjects with subgroups of IBS versus subjects without IBS

**Age, gender and food groups**	**IBS vs non-IBS^A^** **n = 388 vs 4233**		**C-IBS vs non-IBS^B^** **n = 103 vs 4233**		**A-IBS vs non-IBS^C^** **n = 174 vs 4233**		**D-IBS vs non-IBS^D^** **n = 111 vs 4233**	
	**OR (95% CI** ***)***	**p**	**OR (95% CI** ***)***	**p**	**OR (95% CI** ***)***	**p**	**OR (95% CI** ***)***	**p**
Age, years	0.99 (0.97 to 1.00)	0.13	0.98 (0.96 to 1.01)	0.18	0.99 (0.97 to 1.01)	0.33	0.99 (0.97 to 1.01)	0.35
Female	1.16 (0.85 to 1.60)	0.35	2.11 (1.18 to 3.79)	0.012	1.61 (1.02 to 2.52)	0.040	0.74 (0.48 to 1.14)	0.17
Fruits and berries, 100 g/day	0.89 (0.75 to 1.05)	0.17			0.81 (0.64 to 1.03)	0.081		
Vegetables, 100 g/day	1.16 (0.85 to 1.60)	0.13						
Potatoes, 100 g/day	1.10 (0.97 to 1.24)	0.23			0.85 (0.68 to 1.06)	0.15		
Dairy products, portions^1^/day	0.85 (0.78 to 0.93)	0.001	0.90 (0.76 to 1.06)	0.20	0.89 (0.78 to 1.01)	0.075	0.74 (0.63 to 0.86)	< 0.001
Omega 3 fatty acids^2^, g/day	0.89 (0.73 to 1.09)	0.26						
Water, 100 ml/day	1.08 (1.02 to 1.15)	0.002	1.08 (0.97 to 1.20)	0.17	1.11 (1.02 to 1.21)	0.019	1.10 (0.99 to 1.22)	0.068
Juice, 100 ml/day	1.05 (0.94 to 1.16)	0.40						
Carbonated beverages, 100 ml/day	1.07 (1.01 to 1.14)	0.023			1.07 (0.98 to 1.16)	0.13	1.11 (1.00 to 1.22)	0.043
Tea, 100 ml/day	1.05 (1.01 to 1.10)	0.019			1.08 (1.03 to 1.14)	0.003		
Coffee, 100 ml/day	0.99 (0.96 to 1.02)	0.55						
Alcohol, units^3^/day	1.216 (0.89 to 1.66)	0.22			1.18 (0.79 to 1.77)	0.42	1.56 (1.01 to 2.42)	0.045

^1^ Dairy product portions are defined as either 150 ml milk/yoghurt or 20 g (one slice) of cheese.

^2^ Omega 3 fatty acids are calculated from fatty fish and fish oil supplements.

^3^ Units of alcohol are defined as 12.8 g (15 ml) of pure alcohol.

^A^ Adjusted for all variables from table 1. Age, gender, and all food items are shown.

^B^ Adjusted for age, gender, HSCL10, musculoskeletal complaints, triglycerides, HDL, systolic blood pressure, creatinine clearance, dairy products, and water. Age, gender, and food groups included in the analyses are shown.

^C^ Adjusted for age, gender, HSCL10, musculoskeletal complaints, BMI, diastolic blood pressure, triglycerides, HDL, creatinine clearance, number of diseases,fruits and berries, potatoes, milk products, carbonated beverages, tea, and alcohol Age, gender, and food groups included in the analyses are shownz.

^D^ Adjusted for age, gender, HSCL10, musculoskeletal complaints, BMI, systolic blood pressure, HDL, water, milk products, carbonated beverages, and alcohol. Age, gender, and food groups included in the analyses are shown.

In subjects with IBS the severity of symptoms was significantly associated with a high intake of vegetables in subjects with C-IBS and A-IBS and with a low intake of potatoes in subjects with C-IBS (Table 4). In subjects with D-IBS, the severity of symptoms was significantly associated with higher intakes of carbonated beverages, alcohol, and fruits and berries.

**Table 4:** Severity of symptoms in subgroups of IBS associated to age, gender and food groups

**Age, gender and food groups**	**C-IBS^A^n =103**		**A-IBS^B^n = 174**		**D-IBS^C^n = 111**	
	**OR (95% CI** ***)***	**p**	**OR (95% CI** ***)***	**p**	**OR (95% CI** ***)***	**p**
Age, years	1.01 (0.97 to 1.04)	0.80	0.97 (0.93 to 1.00)	0.074	0.96 (0.91 to 1.02)	0.18
Female	0.93 (0.33 to 2.68)	0.90	0.16 (0.07 to 0.37)	< 0.001	0.73 (0.29 to 1.84)	0.51
Fruits and berries, 100 g/day			0.75 (0.43 to 1.31)	0.31	1.93 (1.20 to 3.09)	0.006
Vegetables, 100 g/day	1.97 (1.31 to 2.98)	0.001	1.51 (1.08 to 2.13)	0.016		
Potatoes, 100 g/day	0.53 (0.30 to 0.95)	0.034				
Dairy products, portions^1^/day			0.90 (0.73 to 1.11)	0.33		
Water, 100 ml/day			1.06 (0.91 to 1.24)	0.45		
Juice, 100 ml/day			0.87 (0.66 to 1.15)	0.33		
Carbonated beverages, 100 ml/day	1.16 (0.90 to 1.48)	0.25	0.92 (0.80 to 1.07)	0.28	1.37 (1.12 to 1.67)	0.002
Tea, 100 ml/day			1.07 (0.94 to 1.21)	0.30		
Alcohol, units^2^/day					3.19 (1.62 to 6.28)	0.001

^1^ Dairy product portions are defined as either 150 ml milk/yoghurt or 20 g (one slice) of cheese.

^2^ Units of alcohol are defined as 12.8 g (15 ml) of pure alcohol.

^A^ Adjusted for age, gender, HSCL10, musculoskeletal complaints, BMI, pulse, triglycerides, vegetables, potatoes, and carbonated beverages. Age, gender, and food groups included in the analyses are shown.

^B^ Adjusted for age, gender, HSCL10, musculoskeletal complaints, number of diseases, diastolic blood pressure, systolic blood pressure, triglycerides, creatinine clearance, education, physical activity, fruits and berries, vegetables, milk products, water, juice, tea, and carbonated beverages,. Age, gender, and food groups included in the analyses are shown.

^C^ Adjusted for age, gender, HSCL10, musculoskeletal complaints, number of diseases, BMI, diastolic blood pressure, creatinine clearance, fruits and berries, carbonated beverages, and alcohol. Age, gender, and food groups included in the analyses are shown.

The blood values of omega-3 fatty acids of fish origin (104.7 (37.8)) were significantly correlated with a diet containing omega-3 fatty acids from fatty fish and fish oil supplements (R^2^ = 0.421, p *=* 0.001).

## Discussion

This study is the first one to show that the association between IBS and dietary habits refers to the whole population with IBS and not only those seeking medical advice. There were dietary differences between subjects with and subjects without IBS, between IBS subgroups and there were associations between diet and the severity of symptoms. As this was a cross-sectional study the possibilities for both cause and effect relationships are discussed.

The lower intake of dairy products in IBS and particularly D-IBS compared to subjects without IBS is consistent with some studies that have shown avoidance of milk products and lower intakes of calcium in subjects with IBS compared to controls [10,11]. In contrast, other studies have reported a higher intake of calcium or similar amounts of lactose in subjects with IBS/functional gastrointestinal disorders compared to reference values [5,12]. Some possible explanations of the variation of study results in the intake of dairy products include cultural variation and furthermore the subject’s experience of symptoms connected to the intake of these products and their subsequent avoidance. This fits with the lack of association between the intake of dairy products and the severity of symptoms. Overall recommendations are to avoid lactose-containing products if these are suspected to cause symptoms [13-15]. However, as lactose malabsorption seems to be similarly distributed among subjects with and without IBS [14,16], there may be other components of milk besides lactose that cause symptoms. Casein is a milk protein which coagulates in the stomach and seems to be problematic to digest [17,18]. One study showed associations between milk protein intolerance and D-IBS and A-IBS [19]. Histamine, a component in some cheeses, has also been reported to cause IBS like symptoms, such as diarrhoea and flatulence [20]. Another possible explanation includes IgE- and IgG-mediated food hypersensitivity, of which the role in IBS is inconclusive [15,21-25] The lower intake of dairy products in subjects with IBS could be a cause of IBS, but is probably an effect due to symptoms experienced following ingestion. The cause of these symptoms is unknown.

A higher intake of vegetables was significantly associated with an increased severity of symptoms in C-IBS and A-IBS, and a higher intake of fruits and berries was significantly associated with the severity of symptoms in D-IBS. Other studies support these results [26,27]. Vegetables, such as brussel sprouts and beans, as well as fruits such as prunes are reported to produce gas due to fermentation, and subjects with IBS are reported to handle gas poorly [28]. Some fruits can exert a laxative effect due to their carbohydrate composition which may suggest a cause of symptoms in D-IBS [29]. Salicylates, components of fruits and vegetables, has been suggested to cause gut symptoms in susceptible individuals with gastrointestinal disorders [30]. On the other hand as psyllium and other soluble fibres are shown to reduce symptoms in subjects with IBS [31,32], the higher intake of vegetables seen in C-IBS and A-IBS may be an attempt to soften the stool and reduce the symptoms through the mechanisms of maintenance of a healthy microflora and absorbing water [33]. Thus, the higher intake of fruits, berries and vegetables could be a combination of consequence or cause of symptoms.

The intake of water and tea was significantly higher in A-IBS compared to subjects without IBS and the intake of water and carbonated beverages was particularly high in subjects with D-IBS. Plain water is recommended as a beverage for subjects with IBS [34], while tea may be constipating [35], and coffee, which was not associated with IBS in our study, may be related to diarrhoea [31]. Tea contains salicylates, which may cause gut symptoms [30]. There are recommendations to reduce or to replace caffeine-containing products such as coffee and teas [15,34]. The positive association of IBS with carbonated beverages is consistent with the findings of one study that showed a higher intake of cola in subjects with IBS compared to controls [6]. One study reported more gastrointestinal complaints induced by carbonated beverages among subjects with IBS compared with controls [26]. Thus, the higher intake of carbonated beverages could be a cause of severe symptoms due to intakes of carbonated beverages containing caffeine or other components. Conversely, the intake of carbonated beverages in addition to water could simply be an attempt to replace milk with other fluids or to increase the intake of fluids to manage symptoms as recommended for both constipation- and diarrhoea-related problems [36,37].

Subjects with D-IBS showed the highest intakes of alcohol compared with other subgroups and subjects without IBS, and in this subgroup, there was a significant, positive association between the intake of alcohol and the severity of symptoms. In, contrast, Williams et al. found no alcohol intake differences between IBS subgroups [12]. Supportive of our results, alcohol consumption was associated with gastrointestinal symptoms in other studies [4,38]. Two studies showed support for stool softening properties of alcohol [35,39]. Hey et al. suggested that alcohol may produce osmotic diarrhoea through its high sugar content [40]. The higher intake of alcohol may also just reflect an attempt to relieve severe symptoms with alcohol.

There seems to be issues related to symptoms concerning diary products, fruits and vegetables, carbonated beverages and alcohol in subjects with IBS, but there may be differences between subgroups regards what kind of item causes symptoms. The mentioned food items may be discussed in dietary consultations, and the importance of treating every patient with IBS individually is herby emphasised.

The higher score for psychological distress measured by HSCL10 and the higher score of musculoskeletal complaints in subjects with IBS compared to subjects without IBS are consistent with the results of other studies [41]. The higher percentage of women and the lower age of the IBS population found in unadjusted analyses were not associated with IBS in adjusted analyses, probably due to sex- and age related differences in diet. The more severe symptoms among subjects with D-IBS is consistent with the results of a previous study that found a higher effect from severity of pain in persons with D-IBS and A-IBS compared to those with C-IBS [42]. The higher percentage of men among those with D-IBS is consistent with the results of other studies [43].

### Strengths and limitations

The strengths of this study were the design based on the general population, which reduces the risk for a selection bias, and that the sample size was high, which increases the internal validity of the study and reduces the risk of type II error. However, the response rate was low, which might again induce a selection bias and reduce the external validity. Still, a Norwegian study on non-responders that shares many similarities with our study, such as time period, design, and response rate, found no evidence of major systematic errors [44]. As our study was conducted in the general population, subjects with all grades of severity and subjects who either visited or did not visit doctors were included. Thus, the population with IBS is heterogenic and the results may not be compared with results from studies of merely referred subjects. One other cross-sectional study from the general population was conducted in the Western of Norway a few years prior to our study [45]. The response rate was high (77%). To the extent it was possible to compare the studies; there were no dietary principle differences between this study and our study. The considered p-value < 0.05 might have increased the risk of a type I error, as we had several outcome variables such as IBS yes/no, the severity of symptoms and the subtypes of IBS. The significant and strong correlation between the blood omega-3 fatty acids of fish origin and the self-reported diet of omega-3 fatty acids from fish and food supplements strengthens the validity of the diet data.

The FFQ was unfortunately not validated, however the food questions have been used and published by Lupton [46] and the alcohol questions have been used in some publications [47,48]. As a FFQ is somewhat restricted, it is in general not able to catch the complete diet. The FFQ in our study was additionally limited as it did not include questions about important food groups such as cereals and meat. Different types of fruits or vegetables were not specified and as different fruits and vegetables may exert different effects, the interpretation of the results was difficult. As this was a limited FFQ we were not able to collect data on energy intake or intake of macronutrients. There were also no questions about food frequency. Overestimation of food intake is a common bias in FFQs such as ours. It is unlikely, however, that the population with IBS should overestimate more or less than the population without IBS.

## Conclusions

Our population-based study shows differences in diet in subjects with and without IBS. Some of the differences are more pronounced in subgroups and the diet is associated with the severity of symptoms. The results may have importance for dietary advice. Future research should include all food groups, try to find plausible mechanisms underlying the dietary differences, and differentiate between different subgroups of IBS.

## Abbreviations

IBS: irritable bowel syndrome; C-IBS: constipation-predominant IBS; A-IBS: alternating IBS; D-IBS: diarrhoea-predominant IBS; OR: odds ratio; CI: 95% confidence interval; HSCL10: hopkins symptom checklist 10; FFQ: food frequency questionnaire.

## Competing interests

The authors declare that they have no competing interests.

## Authors’ contributions

PGF wrote the protocol. SCL, SL, and PGF performed statistical analyses. SCL wrote the paper under the supervision of PGF. All authors have read and approved the final manuscript.

## Pre-publication history

The pre-publication history for this paper can be accessed here:

http://www.biomedcentral.com/1471-230X/12/61/prepub
